# Photocatalytic Degradation of Methylene Blue under UV Light Irradiation on Prepared Carbonaceous TiO_2_

**DOI:** 10.1155/2014/415136

**Published:** 2014-06-11

**Authors:** Zatil Amali Che Ramli, Nilofar Asim, Wan N. R. W. Isahak, Zeynab Emdadi, Norasikin Ahmad-Ludin, M. Ambar Yarmo, K. Sopian

**Affiliations:** ^1^Solar Energy Research Institute, Universiti Kebangsaan Malaysia, 43600 Bangi, Selangor, Malaysia; ^2^School of Chemical Science & Food Technology, Faculty of Science and Technology, Universiti Kebangsan Malaysia, 43600 Bangi, Selangor, Malaysia

## Abstract

This study involves the investigation of altering the photocatalytic activity of TiO_2_ using composite materials. Three different forms of modified TiO_2_, namely, TiO_2_/activated carbon (AC), TiO_2_/carbon (C), and TiO_2_/PANi, were compared. The TiO_2_/carbon composite was obtained by pyrolysis of TiO_2_/PANi prepared by in situ polymerization method, while the TiO_2_/activated carbon (TiO_2_/AC) was obtained after treating TiO_2_/carbon with 1.0 M KOH solution, followed by calcination at a temperature of 450°C. X-ray powder diffraction (XRD), transmission electron microscopy (TEM), Fourier transform infrared (FTIR), thermogravimetric analysis (TG-DTA), Brunauer-Emmet-Teller (BET), and UV-Vis spectroscopy were used to characterize and evaluate the prepared samples. The specific surface area was determined to be in the following order: TiO_2_/AC > TiO_2_/C > TiO_2_/PANi > TiO_2_ (179 > 134 > 54 > 9 m^2^ g^−1^). The evaluation of photocatalytic performance for the degradation of methylene blue under UV light irradiation was also of the same order, with 98 > 84.7 > 69% conversion rate, which is likely to be attributed to the porosity and synergistic effect in the prepared samples.

## 1. Introduction

Currently, the rapid industrialization in developing countries has begun to introduce harmful organic pollutants into the water supply. These effluents are also sourced from the textile industry that consumes a large quantity of water in the process of dyeing and washing of fabrics and the release of huge quantities of dyes [1]. Therefore, the development of inexpensive and green methods to treat and purify contaminated water has been the focal subject in technological developments. Among many strategies, photocatalysis is regarded as the most viable one, especially for treatment of contaminants, due to its usage of sunlight to decompose organic pollutants [2–4]. Titanium dioxide or titania (TiO_2_) acts as an agent in this process [5, 6]. However, TiO_2_ is not without its drawback in the context of photocatalysis, such as its high band gap energy 3.2 eV for anatase [7], the limitation of photo response only under UV light region, and its high rate of electron and hole recombination. Attempts to address these problems involve doping, nonmetallic elements doping, surface modification, forming composites with narrow semiconductors, using semiconductor polymer as support, and coating with carbon layers to improve and enhance the photocatalytic activity of titanium dioxide.

Current literature shows that activated carbon is gaining attention as it is capable of modifying TiO_2_ photocatalyst. The work in [8–12] showed that this enhancement is caused by porous structure of activated carbon, which provides a large surface area for photoactive TiO_2_ particle.

The work in [8, 13–15] found the synergistic effect of the mixture of TiO_2_ with activated carbon (AC). The improvement of TiO_2_/AC composite was explained by the high adsorption of the impurities on the surface of activated carbon and their transfer to the TiO_2_ surface. In this study, carbonaceous TiO_2_, namely, TiO_2_/activated carbon (AC), TiO_2_/carbon (C), and TiO_2_/PANi were prepared and evaluated for photocatalytic performance for degradation of methylene blue under UV light irradiation. The carbon and activated carbon/TiO_2_ were synthesized using polyaniline (PANi) as its carbon source.

## 2. Experimental and Characterizations

### 2.1. Preparation of TiO_2_/Polyaniline and TiO_2_/Carbon

TiO_2_/polyaniline (TiO_2_/PANi) was synthesized using TiO_2_ (Sigma Aldrich, 99.9% purity) and aniline hydrochloride (Sigma Aldrich, 99.95%). In order to synthesize TiO_2_/PANi, 0.2 mol cm^−3^ aniline hydrochloride solution was prepared by adding 259 mg aniline hydrochloride to 5 mL deionized water and was vigorously stirred for 10 minutes at a temperature of 60°C. TiO_2_ powder, with various percentages (5%, 10% and 15%), is added to aniline hydrochloride solution being prepared previously. 0.25 mol cm^−3^ ammonium peroxydisulphate solution was prepared by adding 571 mg ammonium peroxydisulphate (Sigma Aldrich, 98% reagent grade) in 5 mL deionized water. While the mixture above was being stirred, the prepared ammonium peroxydisulphate solution was added to the mixture and was stirred for an additional 10 minutes. The polymerization was completed in about 10 minutes. The solution was then left to dry at room temperature for 48 hours. The precipitate powder was then centrifuged and washed with absolute ethanol, followed by distilled water in order to remove unreacted aniline monomer and its corresponding by-products. The product (TiO_2_/PANi) was dried at 65°C for 24 h. The composite that was the result of the process was labeled as TiO_2_/PANi. The product was then pyrolyzed at a temperature of 450°C for 1 hour in nitrogen flow at a heating rate of 10°C min^−1^ to produce titania/carbon (TiO_2_/C).

### 2.2. Chemical Activation of TiO_2_/Carbon

In order to produce porous carbon or the so-called activated carbon, the TiO_2_/C was treated with 1.0 M KOH solution. About 0.3 g TiO_2_/C was mixed with 2.5 mL 1.0 M KOH solution. The mixture was then heated at a temperature of 450°C under nitrogen gas flow for 1 hour. Then the treated sample was washed with deionized water until it was neutral and dried overnight at a temperature of 100°C and was labeled TiO_2_/AC. Since the early photocatalytic performance tests demonstrated better result for the TiO_2_/AC prepared from 15% TiO_2_/PANi, the characterization was done for 15% TiO_2_/PANi and the resulting TiO_2_/C and TiO_2_/AC (hereafter denoted as TiO_2_/PANi, TiO_2_/C, and TiO_2_/AC, resp.).

### 2.3. Characterizations

The functionality groups of the samples were determined using FTIR spectra. The morphology and structure of the samples were determined by field emission scanning electron microscope (FESEM, ZEISS Supra VP55) and transmission electron microscope (JEOL JEM-2100). The specific surface area, pore size, pore volume, and pore diameter of the samples were determined by Brunauer-Emmet-Teller (BET) method [16] using a nitrogen adsorption instrument (Micrometics ASAP 2010). The samples were degassed at 100°C for 24 hours prior to the analysis. Pore size distribution was calculated from the adsorption desorption of the isotherms using the Barret-Joyner-Halenda (BJH) model [17], while the crystallinity of the samples was analyzed using Brucker DB-advance X-ray diffractometer (XRD). The analyses were done under the setting of Cu k*α* radiation at 2 theta, ranging from 10° to 80° for a 1-gram sample. The particle size of the sample was calculated by applying Scherrer's equation. Thermal gravimetric analysis (TGA) with a simultaneous TGA-DTG system (model: Mettler Toledo) has been used for investigation of thermal stability of samples. To reduce the influence of the sample quantity on the analyses, 5 ± 0.2 mg of each sample was used in each analysis with a constant nitrogen (N_2_) flow 50.0 mL min^−1^ maintained throughout the entire process. To minimize possible differences in the moisture content between samples, all TGA samples were equilibrated at 50°C for 5 min before being heated to 700°C at a ramping rate of 5°C min^−1^.

For investigation of prepared samples recyclability, the used catalyst was separated from reaction mixture by filtration. After that, it is washed with distilled water several times and dried in oven to be reused for next reaction cycle. Then they were used for subsequent cycles under similar reaction conditions as carried out by fresh catalyst.

### 2.4. Photocatalytic Experimental

The photocatalytic activities of the samples were evaluated via the photocatalytic oxidation of methylene blue (MB) under UV light irradiation. A 15 watt UV bench lamp was used as a light source. Since the photocatalytic test took into account different MB concentrations (0.05, 0.1, and 0.15 mM) and different irradiation times, it showed better results for 0.05 mM and 90 minutes, respectively, and was selected as the test condition (see Section 3.2).

Prior to illumination, 20 mg photocatalyst was added to the MB solution (20 mL, 0.05 mM). The solution was stirred in the dark for 30 minutes in order to reach MB absorption-desorption equilibrium, which will then allow for the commencement of the photocatalytic reaction. The photocatalyst will then be exposed to the UV lamp for 90 minutes in room temperature. The results after this time period will be evaluated after 90 minutes.

The degradation efficiency of MB was analyzed using UV-Vis spectrometer. Peaks were observed to be present between 600 and 700 nm and were assigned as the absorption of the *π*-system [18], which was indicative of the degradation of MB. According to Beer-Lambert Law, MB's concentration is directly proportional to its absorbance. This makes it possible to determine the oxidation efficiency of MB using the following equation:

(1) $$\begin{array}{c} R=\frac{\left(C_{o}-C_{t}\right)}{C_{o}}\times 100\%, \end{array}$$

where *C*_*o*_ is the initial concentration of MB solution and *C*_*t*_ is concentration of MB during irradiation.

## 3. Results and Discussions

**Figure 1:**
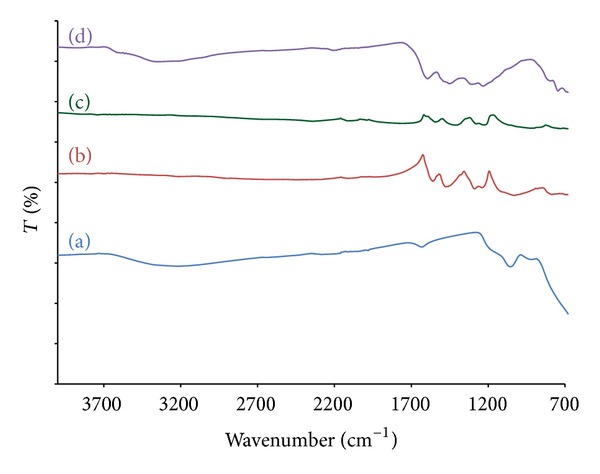
FTIR spectra of (a) TiO_2_, (b) Polyaniline, (c) TiO_2_/PANi, and (d) TiO_2_/AC, respectively.

The existence of polyaniline on the TiO_2_ surface particle was verified by IR spectra. The characteristic bands of TiO_2_, polyaniline (made in the same way for comparison purpose only), TiO_2_/PANi, and TiO_2_/AC are shown in Figures 1(a), 1(b), 1(c), and 1(d), respectively. The FTIR spectrum of polyaniline was clearly observed in TiO_2_/PANi sample. The spectra display the two bands at 1490 and 1585 cm^−1^, which were assigned to benzenoid and quinoide rings. The band at 1300 cm^−1^ is related to C–N stretching of a secondary aromatic amine, while the band at 1140 cm^−1^ is attributed to the vibration mode of the polymer. These bands reveal the existence of PANi in the synthesized TiO_2_/PANi sample. In TiO_2_ and TiO_2_/AC spectra, peaks around 3400 cm^−1^ were assigned to –OH stretching, while those at 1600 cm^−1^ were assigned to –OH vibration. These peaks can be attributed to the adsorbed water from the environment.

**Figure 2:**
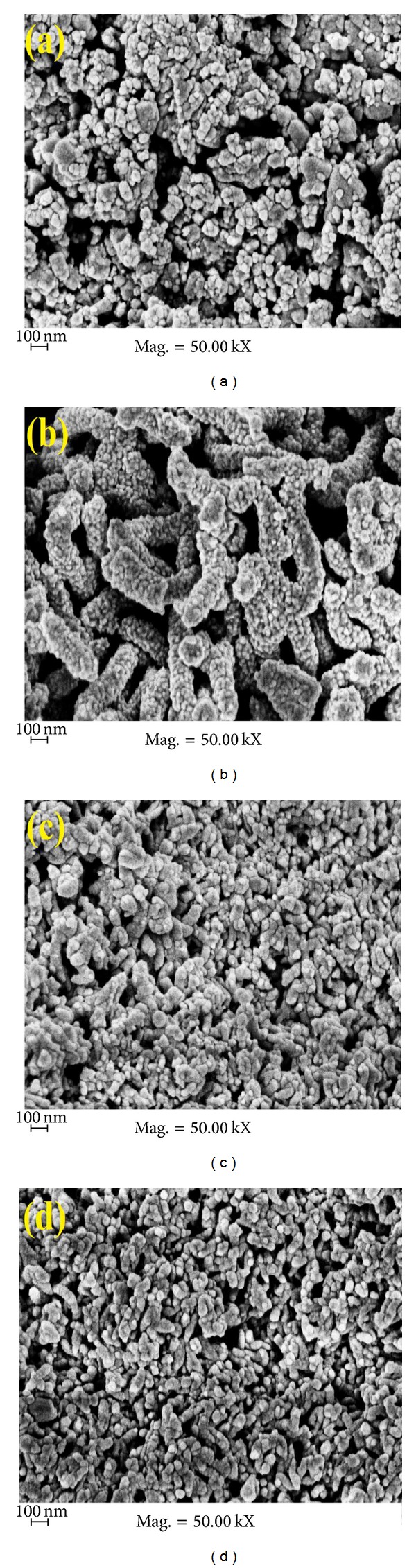
FESEM photographs of (a) TiO_2_ particles, (b) TiO_2_/PANi, (c) TiO_2_/C, and (d) TiO_2_/AC, respectively.

**Figure 3:**
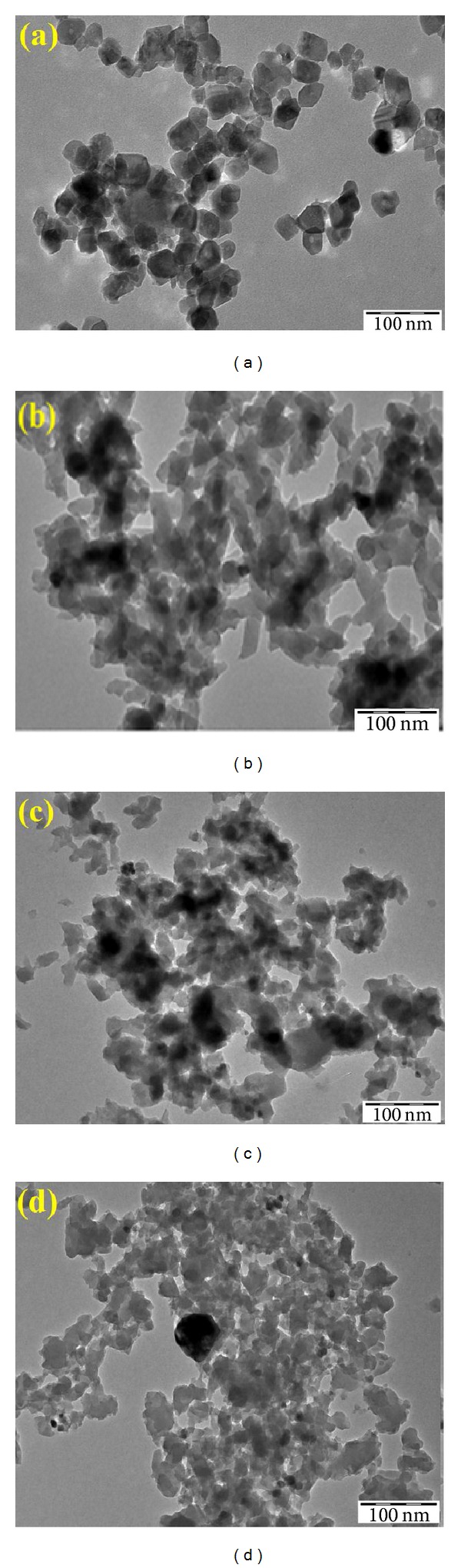
TEM photographs of (a) TiO_2_ particles, (b) TiO_2_/PANi, (c) TiO_2_/C, and (d) TiO_2_/AC, respectively (magnification 45000x).

The morphologies and size of the prepared nanoparticles were studied by variable pressure scanning electron microscope (VPSEM) and transmission electron microscopy (TEM) (Figures 2 and 3).

From the TEM image of Figure 3(a), it was shown that bare TiO_2_ particles are uniform in shape, with sizes ranging from 25 to 30 nm.  Figure 3(b) shows TiO_2_/PANi composite, where the sizes range from 30 to 40 nm. Both TEM and FESEM photographs show that there is no significant difference of particle size of the samples TiO_2_/C and TiO_2_/AC (before and after chemical activation of KOH solution) (40–80 nm). This agrees with previous studies, which reported that the sintering effect did not occur for TiO_2_/carbon after being heated at temperatures below 700°C [19].

**Figure 4:**
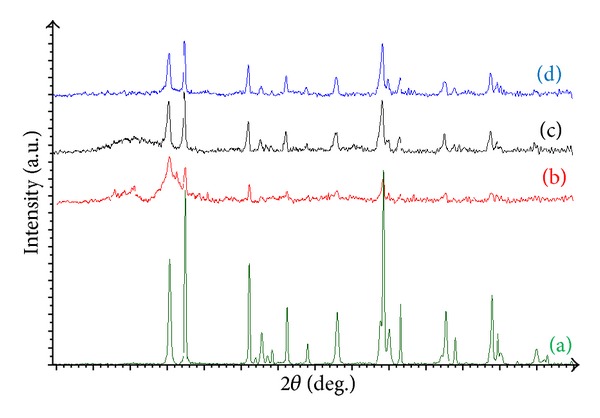
XRD patterns of samples. (a) TiO_2_ particles, (b) TiO_2_/PANi, (c) TiO_2_/C, and (d) TiO_2_/AC, respectively.

The XRD patterns of the prepared samples were shown in Figure 4. The main diffraction peaks were at 25.3°, 37.8°, 48°, 54°, and 62.6°, assigned to the diffraction planes of (1 0 1), (0 0 4), (2 0 0), (1 0 5), and (2 0 4) for anatase (JCPDS number 021-1272), respectively. Other crystal phases corresponding to the peaks at 27.4 and 36.1° were assigned to the diffraction peaks of (1 1 0) and (1 0 1) of rutile (JCPDS number 021-1276), respectively. The XRD patterns revealed the diffraction peaks of a mixture of anatase and rutile in the prepared samples, which is favorable for photocatalytic reactions [20–23]. XRD also showed that the preparation of TiO_2_ composites did not affect the crystal structure of TiO_2_, but the peaks intensity became lower and its shape got wider, especially in the case of TiO_2_/PANi. This can be attributed to certain crystalline structure in PANi overlapping TiO_2_ peaks, but this effect is less pronounced in TiO_2_/C and TiO_2_/AC. The anatase content (*f*(*A*)) was determined from the integrated intensity of the anatase diffraction line (1  0  1),  *I*_*A*_, and that of the rutile diffraction line (1  1  0), *I*_*R*_, using the following equation [24] and the results are listed in Table 1:

(2) $$\begin{array}{c} f\left(A\right)=\frac{1}{1+12.6\left(I_{A}/I_{R}\right)}. \end{array}$$

The *f*(*A*) values showed that the rutile phase is dominant in all of the prepared samples. Besides that, the crystallite size of the samples was calculated by applying the Scherrer equation on the plane (101) diffraction peak of anatase and (110) diffraction peak of rutile, as listed in Table 1. The BET surface area, pore size, micropore area, and volumes are listed in Table 1 as well. The results showed that the specific surface area adhered to the following order: TiO_2_/AC > TiO_2_/C > TiO_2_/PANi >TiO_2_ (179 >134 > 54 > 9 m^2 ^g^−1^). This suggests that the characteristics of the composited catalyst were enhanced, as it provided more active sites for photocatalytic reactions to take place.

**Figure 5:**
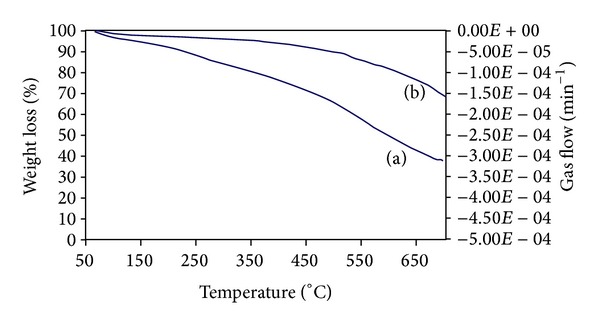
TGA thermogram analysis of (a) TiO_2_/PANi and (b) TiO_2_/AC composite.

The thermal stability of the prepared TiO_2_/PANi and TiO_2_/AC was investigated and the TG-DTA analysis results are shown in Figure 5. The first weight loss took place in the range of temperature 50–120°C for TiO_2_/PANi, which is 6.5% decrease from the original weight. The weight loss is due to the loss of moisture or water content, the evaporation of solvents such as ethanol and small molecules from the polymer matrix [25]. The second loss was determined at temperatures between 310 and 700°C. During this step, the weight loss was attributed to the degradation of polyaniline main chain (about 54%) [26–28]. At a temperature of about 700°C, the PANi molecule was completely decomposed to carbon. The result showed that TiO_2_/AC has significantly improved thermal stabilities compared to TiO_2_/PANi.

### 3.1. Evaluation of Photocatalytic Performance

**Figure 6:**
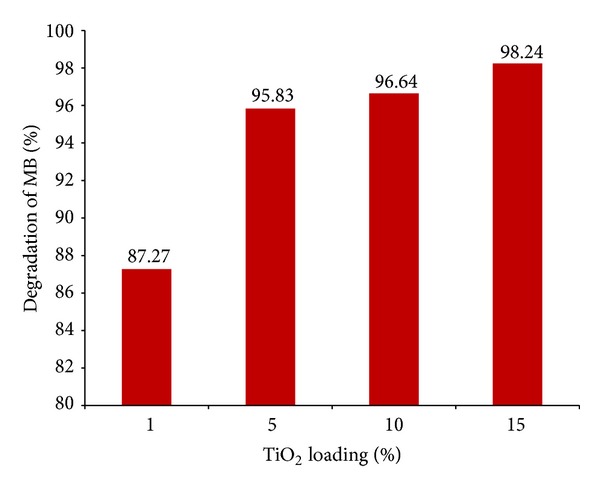
Effect of TiO_2_ loading (%) on degradation of MB (under 90 minutes of UV light irradiation in room temperature).

The degradation of methylene blue (MB) was used in this work in order to evaluate the photocatalytic activity of the prepared samples. The effect of different loading percentages of TiO_2_ in activated carbon on photocatalytic activity has been investigated as well (Figure 6). The results showed that increasing the content of TiO_2_ improves photocatalytic performances. From the tests, it was determined that the 15% TiO_2_ loading was to be further tested.

**Figure 7:**
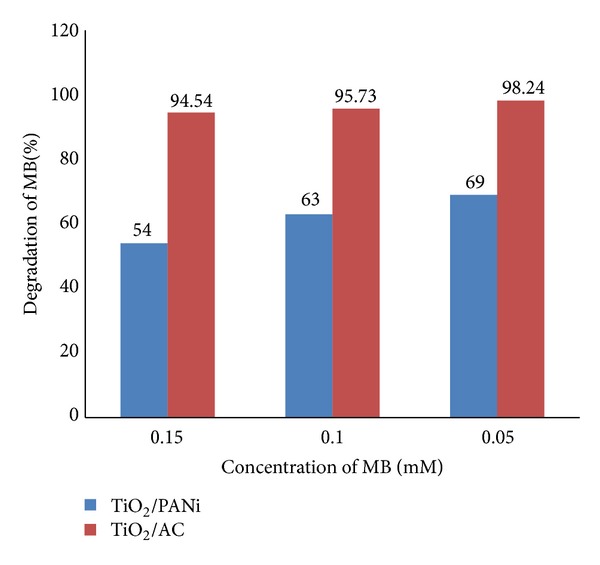
Effect of MB concentration on photocatalytic activity for TiO_2_/PANi and TiO_2_/AC.

It is known that the concentration of methylene blue influences the photocatalytic performance of the samples [29]. Therefore, in this study, the photocatalytic activity of TiO_2_/PANi and TiO_2_/AC (0.05 mM) using different concentrations of MB within 90 minutes irradiation time was studied. It can be seen that TiO_2_/activated carbon demonstrated superior photocatalytic activity for all different concentrations of MB (Figure 7).

**Figure 8:**
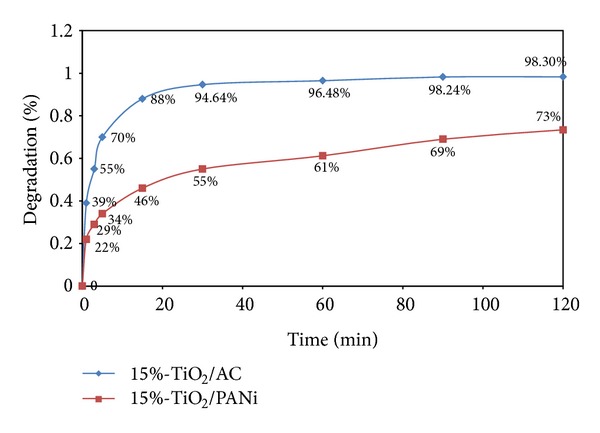
Photocatalytic activity: effect of irradiation time in degradation of MB.

The photocatalytic activity was also evaluated using irradiation time acting as its parameter (Figure 8). The result show rapid increase in MB degradation from 0 to 30 minutes which becomes slower after 30 minutes for both samples. The much higher MB degradation rate for TiO_2_/AC proves its better performance compared to TiO_2_/PANi. TiO_2_/AC showed almost complete degradation of MB after 90 minutes of irradiation.

**Figure 9:**
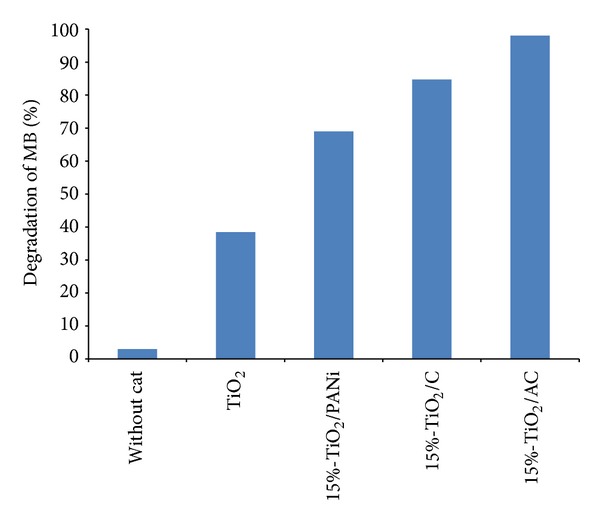
Photocatalytic degradation of methylene blue for bare TiO_2_, TiO_2_/PANi, TiO_2_/C, and TiO_2_/AC (0.05 mM MB and 90 min irradiation time).

Figure 9 presents the result of the degradation of MB for bare TiO_2_, TiO_2_/PANi, TiO_2_/C, and TiO_2_/AC. The experiment was carried out in 90-minute irradiation, and the concentration of methylene blue was 0.05 mM for all samples. The degradation of MB without catalyst was used as a reference.

The results can be explained using texture properties of the prepared samples, as shown in Table 1. The specific surface area, micropore area, and volume adhered to the trend TiO_2_/AC > TiO_2_/C > TiO_2_/PANi >TiO_2_, which is similar to photocatalytic performance. This is attributed to the higher porosity of the activated carbon compered to others, which provide high adsorption capacity and more active sites for the reacting species during chemical reaction, which agrees with previous studies [8, 9, 19, 30–32]. It was noted that the surface area of TiO_2_/AC is larger compared to the ones reported in the literature, which used polystyrene (PS) as a carbon source [33]. It also shows that PANi is a better source for the preparation of highly porous activated carbon.

On top of the synergistic effect for the mixture of TiO_2_ with activated carbon and highly porous structures, the high photocatalytic performance could be the result of the mixture of anatase and rutile phase of TiO_2_ in this work (Table 1). This can be explained by the synergic effect between anatase and rutile in TiO_2_ particles that are approximately close to each other, thus enhancing the activity [34]. This finding is in agreement with other studies reporting that the photocatalytic activity was enhanced when anatase and rutile were mixed [34, 35].

### 3.2. Recyclability of TiO_2_/AC

**Figure 10:**
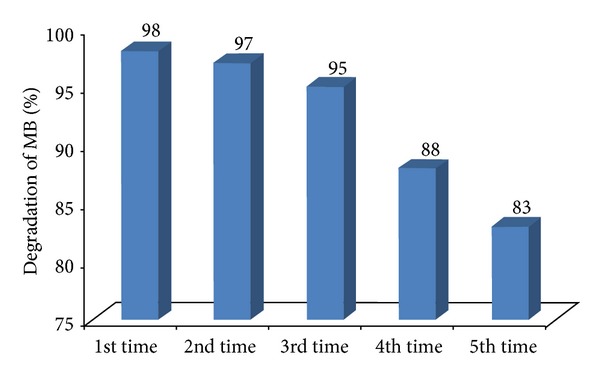
Recyclability of TiO_2_/AC was carried out for 5 cycles used.

Recyclability test was carried out for TiO_2_/AC to up to five cycles. The results are depicted in Figure 10, demonstrating good recyclability for the prepared composite.

## 4. Conclusion

Regarding the possibility of photocatalytic improvement using a composite of TiO_2_, a different form of modified TiO_2_, namely, TiO_2_/activated carbon (AC), TiO_2_/Carbon (C), and TiO_2_/PANi, were synthesized and characterized, and their catalytic performance have been studied. The effect of TiO_2_ loading, irradiation time, and MB concentration has been studied. TiO_2_/AC showed the highest porosity and photocatalytic performance compared to other composites. On top of considering the synergistic effect for the mixture of TiO_2_ with activated carbon and its high porosity, this high performance can also be attributed to the anatase and rutile mixture. Meanwhile, PANi demonstrated better AC source compared to previous works, and the recyclability test demonstrated excellent performance for the synthesized composite.

## Figures and Tables

**Table 1:** Texture properties of the TiO_2_, TiO_2_/PANi, TiO_2_/C, and TiO_2_/AC composites.

Physical properties	TiO_2_	TiO_2_/PANi	TiO_2_/C	TiO_2_/AC
Surface area (m^2^/g)	8.55	53.73	134	178.57
Pore size (nm)	9.48	13.8	9.26	6.79
Micropore area (m^2^/g)	3.34	8.22	29.33	76.62
Micropore volume (cm^3^/g)	0.001	0.004	0.015	0.037
Crystallite size^a^ (nm) anatase	25	14	—	11
Crystallite size^a^ (nm) rutile	48	19	—	20
*f*(*A*)^b^	0.135	0.061	0.087	0.092

^a^Calculated by applying Scherrer's equation.

^b^Anatase content calculated using (2).

## References

[1] Hussain S, Maqbool Z, Ali S (2013). Biodecolorization of reactive black-5 by a metal and salt tolerant bacterial strain Pseudomonas sp. RA20 isolated from Paharang drain effluents in Pakistan. *Ecotoxicology and Environmental Safety*.

[2] Zhao J, Chen C, Ma W (2005). Photocatalytic degradation of organic pollutants under visible light irradiation. *Topics in Catalysis*.

[3] Bahnemann D (2004). Photocatalytic water treatment: solar energy applications. *Solar Energy*.

[4] Gaya UI, Abdullah AH (2008). Heterogeneous photocatalytic degradation of organic contaminants over titanium dioxide: a review of fundamentals, progress and problems. *Journal of Photochemistry and Photobiology C: Photochemistry Reviews*.

[5] Hoffmann MR, Martin ST, Choi W, Bahnemann DW (1995). Environmental applications of semiconductor photocatalysis. *Chemical Reviews*.

[6] Carp O, Huisman CL, Reller A (2004). Photoinduced reactivity of titanium dioxide. *Progress in Solid State Chemistry*.

[7] Tachikawa T, Fujitsuka M, Majima T (2007). Mechanistic insight into the TiO_2_ photocatalytic reactions: design of new photocatalysts. *Journal of Physical Chemistry C*.

[8] Liu SX, Chen XY, Chen X (2007). A TiO_2_/AC composite photocatalyst with high activity and easy separation prepared by a hydrothermal method. *Journal of Hazardous Materials*.

[9] Velasco LF, Parra JB, Ania CO (2010). Role of activated carbon features on the photocatalytic degradation of phenol. *Applied Surface Science*.

[10] Isahak WNRW, Hisham MWM, Yarmo MA (2013). Highly porous carbon materials from biomass by chemical and carbonization method: a comparison study. *Journal of Chemistry*.

[11] Torimoto T, Okawa Y, Takeda N, Yoneyama H (1997). Effect of activated carbon content in TiO_2_-loaded activated carbon on photodegradation behaviors of dichloromethane. *Journal of Photochemistry and Photobiology A: Chemistry*.

[12] Tryba B (2008). Increase of the photocatalytic activity of TiO_2_ by carbon and iron modifications. *International Journal of Photoenergy*.

[13] Matos J, Laine J, Herrmann J (1999). Association of activated carbons of different origins with titania in the photocatalytic purification of water. *Carbon*.

[14] Araña J, Doña-Rodríguez JM, Tello Rendón E (2003). TiO_2_ activation by using activated carbon as a support: part I. Surface characterisation and decantability study. *Applied Catalysis B: Environmental*.

[15] Araña J, Doña-Rodríguez JM, Tello Rendón E (2003). TiO_2_ activation by using activated carbon as a support: part II. Photoreactivity and FTIR study. *Applied Catalysis B: Environmental*.

[16] Brunauer S, Emmett PH, Teller E (1938). Adsorption of gases in multimolecular layers. *Journal of the American Chemical Society*.

[17] Barrett EP, Joyner LG, Halenda PP (1951). The determination of pore volume and area distributions in porous substances. I. Computations from nitrogen isotherms. *Journal of the American Chemical Society*.

[18] Liu G, Wu T, Zhao J, Hidaka H, Serpone N (1999). Photoassisted degradation of dye pollutants. 8. Irreversible degradation of alizarin red under visible light radiation in air-equilibrated aqueous TiO_2_ dispersions. *Environmental Science and Technology*.

[19] Tryba B, Morawski AW, Inagaki M (2003). Application of TiO_2_-mounted activated carbon to the removal of phenol from water. *Applied Catalysis B: Environmental*.

[20] Ovenstone J (2001). Preparation of novel titania photocatalysts with high activity. *Journal of Materials Science*.

[21] Moon J, Takagi H, Fujishiro Y, Awano M (2001). Preparation and characterization of the Sb-doped TiO_2_ photocatalysts. *Journal of Materials Science*.

[22] Arbuj SS, Hawaldar RR, Mulik UP, Wani BN, Amalnerkar DP, Waghmode SB (2010). Preparation, characterization and photocatalytic activity of TiO_2_ towards methylene blue degradation. *Materials Science and Engineering B: Solid-State Materials for Advanced Technology*.

[23] Toyoda M, Nanbu Y, Nakazawa Y, Hirano M, Inagaki M (2004). Effect of crystallinity of anatase on photoactivity for methyleneblue decomposition in water. *Applied Catalysis B: Environmental*.

[24] Spurr RA (1957). Quantitative analysis of anatase-rutile mixtures with an X-ray diffractometer. *Analytical Chemistry*.

[25] Elsayed AH, Mohy Eldin MS, Elsyed AM, Abo Elazm AH, Younes EM, Motaweh HA (2011). Synthesis and properties of polyaniline/ferrites nanocomposites. *International Journal of Electrochemical Science*.

[26] Deng J, Ding X, Zhang W (2002). Magnetic and conducting Fe_3_O_4_-cross-linked polyaniline nanoparticles with core-shell structure. *Polymer*.

[27] Asim N, Radiman S, Yarmo MAB (2008). Preparation and characterization of core-shell polyaniline/V_2_O_5_ nanocomposite via microemulsion method. *Materials Letters*.

[28] Lee S, Chen Y, Ho C, Chang C, Hong Y (2007). A study on synthesis and characterization of the core-shell materials of Mn_1-x_Zn_x_Fe_2_O_4_-polyaniline. *Materials Science and Engineering B: Solid-State Materials for Advanced Technology*.

[29] Mohamed RM, Mkhalid IA, Baeissa ES, Al-Rayyani MA (2012). Photocatalytic degradation of methylene blue by Fe/ZnO/SiO_2_ nanoparticles under visiblelight. *Journal of Nanotechnology*.

[30] Li Y, Zhang S, Yu Q, Yin W (2007). The effects of activated carbon supports on the structure and properties of TiO_2_ nanoparticles prepared by a sol-gel method. *Applied Surface Science*.

[31] Wang X, Hu Z, Chen Y, Zhao G, Liu Y, Wen Z (2009). A novel approach towards high-performance composite photocatalyst of TiO_2_ deposited on activated carbon. *Applied Surface Science*.

[32] Zhang X, Zhou M, Lei L (2005). Preparation of photocatalytic TiO_2_ coatings of nanosized particles on activated carbon by AP-MOCVD. *Carbon*.

[33] Lubis S, Yuliati L, Lee SL, Sumpono I, Nur H (2012). Improvement of catalytic activity in styrene oxidation of carbon-coated titania by formation of porous carbon layer. *Chemical Engineering Journal*.

[34] Ohno T, Tokieda K, Higashida S, Matsumura M (2003). Synergism between rutile and anatase TiO_2_ particles in photocatalytic oxidation of naphthalene. *Applied Catalysis A: General*.

[35] Wu C, Yue Y, Deng X, Hua W, Gao Z (2004). Investigation on the synergetic effect between anatase and rutile nanoparticles in gas-phase photocatalytic oxidations. *Catalysis Today*.

